# Phospholipase Cζ, the Molecular Spark of Fertilization and Male Infertility: Insights from Bench to Bedside

**DOI:** 10.3390/medicina61060963

**Published:** 2025-05-23

**Authors:** Aris Kaltsas, Maria-Anna Kyrgiafini, Zissis Mamuris, Fotios Dimitriadis, Athanasios Zachariou, Michael Chrisofos, Nikolaos Sofikitis

**Affiliations:** 1 Third Department of Urology, Attikon University Hospital, School of Medicine, National and Kapodistrian University of Athens, 12462 Athens, Greece; ares-kaltsas@hotmail.com (A.K.); mchrysof@med.uoa.gr (M.C.); 2 Laboratory of Genetics, Comparative and Evolutionary Biology, Department of Biochemistry and Biotechnology, University of Thessaly, 41500 Larissa, Greece; mkyrgiafini@uth.gr (M.-A.K.); zmamur@uth.gr (Z.M.); 3 Department of Urology, Faculty of Medicine, School of Health Sciences, Aristotle University of Thessaloniki, 54124 Thessaloniki, Greece; helabio@yahoo.gr; 4 Laboratory of Spermatology, Department of Urology, Faculty of Medicine, School of Health Sciences, University of Ioannina, 45110 Ioannina, Greece; azachariou@uoi.gr

**Keywords:** phospholipase C zeta, oocyte activation, male infertility, intracytoplasmic sperm injection, globozoospermia, total fertilization failure, calcium oscillations, assisted oocyte activation, gene therapy, PLCZ1 mutations

## Abstract

Phospholipase C zeta (PLCζ) has emerged as a pivotal sperm-specific factor responsible for triggering oocyte activation, a process essential for successful fertilization and early embryogenesis. A narrative review was conducted to examine the molecular architecture and biochemical features of PLCζ, with particular emphasis on how its distinctive structural domains facilitate the hydrolysis of phosphatidylinositol 4,5-bisphosphate (PIP_2_) and the induction of calcium (Ca^2^^+^) oscillations in the oocyte. Notably, PLCζ exhibits unique sensitivity to basal Ca^2^^+^ levels and the capacity to sustain repetitive intracellular Ca^2^^+^ transients that drive meiotic progression and block polyspermy. Clinically, PLCζ deficiency—whether caused by genetic mutations, reduced expression, or improper localization—represents a unifying explanation for certain forms of male infertility, including total fertilization failure (TFF) following intracytoplasmic sperm injection (ICSI). Globozoospermia is a prime example; this condition is characterized by round-headed sperm devoid of acrosomes and exhibiting significantly reduced or absent PLCζ and often results in fertilization failure. Diagnostic methods such as immunofluorescence, Western blotting, and the mouse oocyte-activation test collectively support the identification and characterization of PLCζ-related defects, while genetic testing for mutations in the *PLCZ1* gene has proven valuable for identifying hereditary causes of sperm-borne oocyte-activation deficiency (OAD). Therapeutic approaches range from assisted oocyte activation (AOA) with calcium ionophores to emerging interventions that introduce functional PLCζ protein or mRNA directly into the oocyte. These advancements demonstrate the rapid translation of foundational discoveries into clinically actionable interventions. Future investigations are poised to refine diagnostic assays, standardize measurement protocols, and explore the potential of gene therapy or CRISPR/Cas9-mediated correction for heritable PLCζ abnormalities. By addressing both the molecular basis and translational applications of PLCζ, recent findings underscore its indispensable role in fertility care and lay out a path toward further innovation in assisted reproductive technologies.

## 1. Introduction

Infertility is a global health issue affecting approximately 8–12% of couples of reproductive age [1]. In nearly half of these cases, a male factor is identified as the sole cause or as a contributing component [2]. Male infertility is a complex condition arising from diverse etiologies including genetic abnormalities, hormonal imbalances, anatomical defects, and impairments in sperm production or function [3]. Assisted reproductive technologies (ART), such as in vitro fertilization (IVF) and especially intracytoplasmic sperm injection (ICSI), have revolutionized the treatment of infertility for many causes. ICSI, which involves the direct injection of a single sperm into the oocyte cytoplasm, bypasses many barriers to fertilization and now accounts for roughly two thirds of ART treatments [4,5]. However, even with ICSI, complete failure of fertilization can still occur in a minority of cases. Total fertilization failure (TFF) is observed in ~2–4% of ICSI attempts despite the use of normal-appearing gametes [4,6]. These failures have drawn attention to the importance of functional sperm components that go beyond standard parameters like count, motility, and morphology. The leading explanation for such cases is oocyte-activation deficiency (OAD)—a failure of the injected sperm to trigger the egg’s activation program [6]. Oocyte activation is a crucial event marked by a series of calcium (Ca^2^^+^) oscillations in the egg, which are required for the resumption of meiosis, for cortical granule exocytosis, which prevents polyspermy, and for the initiation of embryonic development [7].

Researchers have long hypothesized the existence of a sperm-derived “oocyte-activation factor”, and over the past two decades, phospholipase C zeta (PLCζ) has been identified as this key sperm factor. PLCζ, a sperm-specific enzyme, is delivered to the oocyte upon sperm–oocyte fusion (or sperm injection in ICSI) and triggers the characteristic Ca^2^^+^ oscillations required for successful fertilization [7]. The first direct evidence came from Yoon et al. (2008), who showed that human spermatozoa lacking PLCζ failed to induce any Ca^2^^+^ release in the oocyte and could not initiate embryo development [8]. Since that landmark finding, numerous studies have confirmed that insufficient or dysfunctional PLCζ in spermatozoa is strongly associated with failed fertilization and certain forms of male infertility [9]. Thus, beyond the traditional view of sperm as mere carriers of paternal DNA, current insights highlight the multifaceted contribution of the sperm to successful fertilization and early embryogenesis.

This review aims to provide a focused overview of PLCζ. Now that it is recognized as the molecular “spark” of fertilization, PLCζ has become central to understanding certain forms of male infertility, particularly in cases of fertilization failure after ICSI. By exploring its structure, function, and clinical implications, this review highlights how insights into PLCζ bridge the gap from bench to bedside, supporting improved diagnostic and therapeutic approaches in reproductive medicine.

## 2. Molecular and Functional Overview

### 2.1. Structure, Biochemical Features, Expression and Localization of PLCζ

PLCζ is a sperm-specific phosphoinositide phospholipase C enzyme and is the smallest known mammalian PLC isoform (approximately 70–75 kDa) [10]. It consists of four tandem EF-hand domains at the N-terminus, which are followed by the X and Y catalytic domains (separated by a short linker region) and a C2 domain at the C-terminus [11,12]. Notably, PLCζ lacks a pleckstrin homology (PH) domain and Src homology (SH) domains [13,14], distinguishing it from somatic PLC isoforms and reflecting its unique mode of action within the oocyte cytoplasm, where enzymes must be small and diffusive.

Each structural element of PLCζ has a defined role in its function. The EF-hand motifs (calcium-binding domains) confer sensitivity to Ca^2^^+^, even at basal Ca^2+^ levels in the egg cytoplasm, and are implicated in the proper localization of PLCζ after it enters the oocyte. Specifically, according to mouse studies, the EF-hand domain is required for PLCζ’s translocation into the oocyte nucleus (perhaps as part of cell-cycle progression) [15]. The X and Y domains form the catalytic core of PLCζ and are responsible for its enzymatic activity. Together, they create the active site that binds to the substrate phosphatidylinositol 4,5-bisphosphate (PIP_2_), which is located in intracellular membranes. A cluster of basic amino acid residues within these domains facilitates electrostatic interactions with the negatively charged phosphate groups of PIP_2_, thereby positioning the enzyme to hydrolyze PIP_2_ efficiently [13]. The intervening XY-linker region is highly positively charged, which may help target PLCζ to negatively charged membrane surfaces in the oocyte, thereby contributing to its membrane-targeting capability [16]. At the C-terminus, the C2 domain binds to phospholipids and plays a role in anchoring PLCζ to intracellular vesicle membranes. It also appears to be important for the proper initiation and propagation of Ca^2^^+^ oscillations [17]. Despite this extensive knowledge of its domain architecture, the full 3D structure of PLCζ is not yet resolved. Determining its full structure would further illuminate how domain interactions enable its unique enzymatic activity and function during fertilization [18].

PLCζ mRNA has been detected across various stages of spermatogenesis, in spermatocytes as well as in round and elongating spermatids, with the exact expression timing differing between species [19,20,21]. Interestingly, studies in mice have also identified PLCζ transcripts within the epididymis, suggesting possible epididymal contributions to PLCζ synthesis or post-transcriptional modification [22]. In mature spermatozoa, PLCζ protein typically localizes to the equatorial segment of the sperm head, strategically positioning it for efficient delivery into the oocyte cytoplasm during fertilization [23]. Nonetheless, localization patterns vary across species. PLCζ has been reported in the acrosomal, post-acrosomal, equatorial, and mid-piece regions of sperm cells from different species [24]. In human sperm, localization predominantly occurs within the acrosomal and equatorial segments [25].

Studies investigating potential changes in PLCζ levels during sperm capacitation consistently indicate that there is no net de novo increase in total PLCζ protein content, as sperm cells are transcriptionally and translationally inactive during this stage. Instead, evidence from multiple species demonstrates a dynamic redistribution of existing PLCζ within the sperm head [26]. In humans, PLCζ initially localizes to both the acrosomal and post-acrosomal regions, and following capacitation and particularly after the acrosome reaction, it becomes concentrated in the equatorial/post-acrosomal region, positioning it optimally for subsequent release into the oocyte at fertilization [26,27]. Mouse studies have shown similar relocation dynamics; capacitation triggers a redistribution of PLCζ away from the acrosome toward the post-acrosomal region without changing total protein levels, reflecting relocalization rather than new synthesis [27,28]. Likewise, research in bull sperm revealed that during capacitation, PLCζ migrates toward the sperm-head periphery in association with the actin cytoskeleton and is partially released upon the acrosome reaction, again confirming redistribution rather than new protein synthesis [29]. Collectively, these findings across species suggest that capacitation induces post-translational modifications or interactions that reposition PLCζ within the sperm, rather than increase its overall quantity.

### 2.2. Mechanism of Action During Fertilization

During normal fertilization or ICSI, once the sperm fuses with or is injected into the oocyte, PLCζ is released from the sperm head into the ooplasm to perform its function [30]. PLCζ’s enzymatic action is to hydrolyze PIP_2_—a membrane phospholipid present in the egg—into two second messengers: inositol 1,4,5-trisphosphate (IP_3_) and diacylglycerol (DAG) [31]. IP_3_ rapidly binds to IP_3_ receptors in the endoplasmic reticulum (ER) of the oocyte, causing Ca^2^^+^ release from the ER stores [32]. Meanwhile, DAG remains in the membrane and activates protein kinase C (PKC) and other downstream signaling pathways [33]. The sudden increase in cytosolic Ca^2^^+^ initiates a wave of calcium oscillations—repetitive spikes in Ca^2^^+^ concentration that typically last for several hours. These Ca^2^^+^ oscillations represent the hallmark event of oocyte activation and are essential for downstream developmental events. PLCζ is remarkably potent in inducing these oscillations, distinguishing itself from other PLC isoforms, due to its unique biochemical features, high sensitivity to Ca^2^^+^, and effective functionality within the egg environment [34]. Functionally, PLCζ acts as a soluble, diffusive spark: once introduced into the oocyte cytoplasm, it continuously generates IP_3_, thereby sustaining Ca^2^^+^ release throughout the cell [35].

Importantly, a single spermatozoon contains sufficient PLCζ molecules to induce and maintain prolonged Ca^2^^+^ oscillations lasting several hours during fertilization [10]. Experimental evidence has demonstrated that microinjection of purified recombinant PLCζ protein, or even cRNA encoding PLCζ, in remarkably low quantities—ranging from approximately one femtogram (fg) (~10^3^–10^4^ molecules) [36] up to 40–80 fg (~10^5^ molecules) [10]—can replicate robust, fertilization-like Ca^2^^+^ transients within the oocyte, ultimately supporting embryo development [37]. Such extraordinary potency aligns closely with the naturally occurring quantity of PLCζ delivered by a single sperm. Further supporting this, experiments using genetically modified PLCζ-knockout mice revealed that eggs fertilized by PLCζ-deficient sperm fail to exhibit typical sustained Ca^2^^+^ oscillations and display significantly compromised activation, characterized by only a few, delayed Ca^2^^+^ spikes [38]. These findings underscore that sperm-derived PLCζ is essential for the normal pattern of repetitive Ca^2^^+^ signaling, egg activation, and successful embryonic development [39].

**Figure 1:**
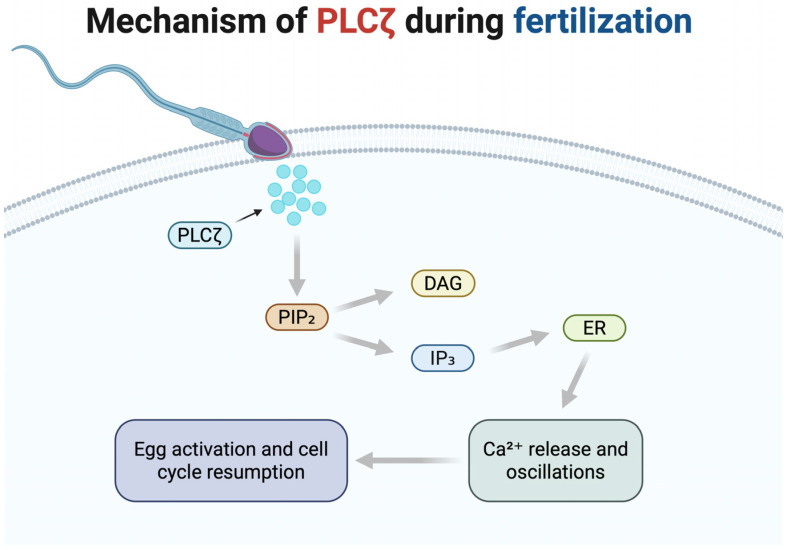
PLCζ-induced mechanism of oocyte activation during fertilization. Created in BioRender. Kaltsas, A. (2025) https://BioRender.com/ha3nu4b (accessed on 21 May 2025).

The sequential steps of PLCζ activity, from its release into the ooplasm to the initiation of calcium oscillations, are illustrated in Figure 1.

The calcium oscillations initiated by PLCζ activate multiple pathways in the egg that collectively constitute oocyte activation. One major consequence is the resumption and completion of meiosis. The Ca^2^^+^ spikes activate calcium/calmodulin-dependent protein kinase II (CaMKII), which phosphorylates and inhibits the cytostatic factor early mitotic inhibitor 2 (EMI2). EMI2 normally maintains meiotic arrest by inhibiting the anaphase-promoting complex/cyclosome (APC/C). Its phosphorylation and subsequent degradation relieve the inhibition of APC/C, allowing for the degradation of cyclin B1 (CNB1), a critical regulatory subunit of the maturation-promoting factor (MPF) [40]. This lifts the metaphase arrest, enabling the oocyte to proceed through anaphase, extrude the second polar body, and form the female pronucleus. Concurrently, Ca^2^^+^/PKC signaling prompts cortical granule exocytosis, a reaction in which the egg releases enzymes that modify the zona pellucida to prevent additional sperm from binding; this establishes the block to polyspermy [41]. Ca^2^^+^ waves also facilitate the inactivation of mitogen-activated protein kinase (MAPK), another essential step required for the formation of the male and female pronuclei. MAPK inactivation removes additional inhibitory controls, thus supporting the formation and subsequent fusion of male and female pronuclei, an event critical to successful embryonic development [40]. In sum, PLCζ’s activity in the egg triggers a cascade: Ca^2^^+^ surges lead to cell-cycle release, polyspermy prevention, and pronuclear formation, which are all hallmarks of successful fertilization and the initiation of embryogenesis [18]. Without PLCζ (or an equivalent Ca^2^^+^ trigger), the oocyte would remain stuck in metaphase and fail to develop even with the sperm’s DNA inside. This underlines why PLCζ is called the molecular spark of life: it ignites the series of biochemical events by which a single-celled zygote is born from the quiescent oocyte and sperm.

## 3. Clinical Relevance

### 3.1. PLCζ Deficiency and Male Infertility

Defects in PLCζ, including mutations, reduced expression, or abnormal localization within spermatozoa, provide a unifying explanation for many cases of male infertility where fertilization fails despite sperm reaching the oocyte (particularly in ICSI) [8]. Several clinical studies have documented mutations in the *PLCZ1* gene in infertile men who experience recurrent fertilization failures [42,43,44,45]. Such mutations typically result in altered PLCζ enzymatic activity, defective localization within the sperm, or impaired capacity to induce calcium oscillations within the oocyte, leading to oocyte-activation deficiency (OAD) and total fertilization failure (TFF). In such cases, the eggs remain unfertilized (stuck in metaphase II) because the sperm, although it penetrated the oocyte, could not trigger activation. Notably, this scenario can occur even in men with otherwise normal semen parameters—representing a hidden cause of male-factor infertility. For example, studies have documented normozoospermic men with repeated ICSI failure who were found to have dramatically low PLCζ levels in their sperm [46]. A notable case report described a man with unexplained fertilization failure whose sperm showed 40–80% lower PLCζ protein levels (measured by Western blot) compared to fertile controls [47]. His sperm induced also virtually no Ca^2^^+^ release in a mouse oocyte assay [47]. Such findings confirm that an occult PLCζ deficiency can underlie infertility even when routine semen analysis appears normal. Identifying these cases is clinically important, as they may benefit from specific interventions.

Beyond idiopathic cases, certain defined male-infertility conditions are strongly linked to PLCζ problems, with globozoospermia being the clearest example. This rare disorder is characterized by round-headed spermatozoa that completely lack an acrosome and is almost invariably associated with oocyte-activation failure [48]. Multiple studies have shown that men with globozoospermia have significantly lower levels of PLCZ1 mRNA and PLCζ protein in their sperm compared to fertile men [49]. In one analysis, globozoospermic patients with the common *DPY19L2* gene deletion exhibited virtually undetectable levels of PLCζ in their sperm, whereas patients with partial globozoospermia (with some acrosomal structures) had low but measurable levels of PLCζ [50]. The absence of PLCζ explains why eggs injected with globozoospermic sperm typically fail to activate; sperm lack the “spark” required to initiate Ca^2^^+^ oscillations. Consequently, globozoospermia patients experience near-universal TFF with standard ICSI. Thus, PLCζ deficiency is considered a hallmark of the globozoospermia phenotype.

**Figure 2:**
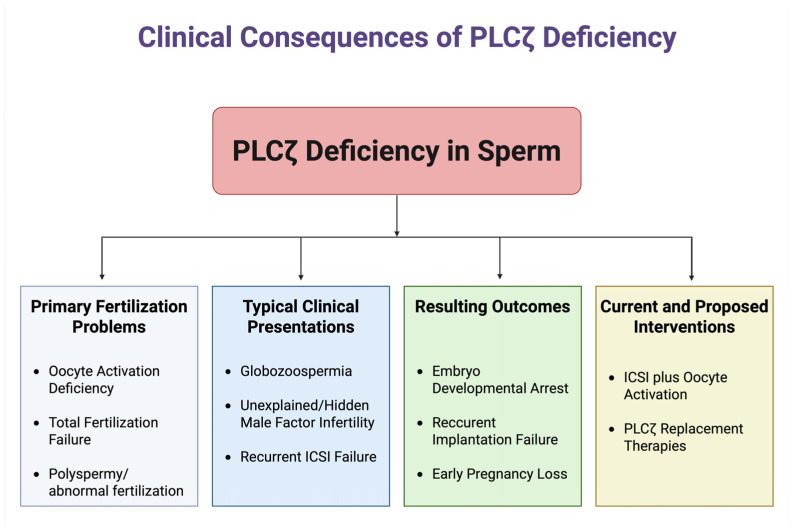
Clinical manifestations of PLCζ deficiency in human sperm. Created in BioRender. Kaltsas, A. (2025) https://BioRender.com/vvmrp86 (accessed on 21 May 2025).

More broadly, other forms of teratozoospermia and mixed male-factor infertility can involve compromised PLCζ. Sperm morphological defects that affect the sperm head or perinuclear theca (where PLCζ resides) may affect the content or localization of PLCζ. For instance, men with severe oligoasthenoteratozoospermia (OAT) have been found to exhibit a lower proportion of PLCζ-positive sperm and atypical patterns of PLCζ localization [51]. In a study of 25 men with OAT, only ~63.4% of sperm were PLCζ-positive, versus ~86.7% in fertile controls, and mislocalization to the post-acrosomal region was frequently observed [52]. In OAT samples, PLCζ is often mislocalized to the sperm tail or post-acrosomal region, and overall PLCζ levels per sperm are reduced [7]. Such defects correlate with lower fertilization rates. There is also evidence linking indicators of sperm health to PLCζ: one study noted that the percentage of PLCζ-expressing sperm was negatively correlated with DNA-fragmentation levels and was significantly reduced in men with high levels of sperm DNA fragmentation [53]. Additionally, certain testicular conditions can impact PLCζ production. For example, in a cohort of 35 infertile men with grade II–III varicocele, PLCZ1 transcript and PLCζ protein levels in sperm were significantly reduced—by approximately 50%—compared to those in 20 fertile men, as measured by RT-PCR and Western blot, likely due to oxidative stress impairing spermatogenic cells in the testes [54]. These findings reinforce that PLCζ is a critical sperm factor; when sperm formation or function is disrupted, PLCζ often becomes deficient, leading to a loss of fertilization competence. Identifying a PLCζ deficiency in an infertile man reclassifies the diagnosis as a sperm factor-mediated OAD, which has important implications for management. An overview of the clinical manifestations and reproductive consequences associated with PLCζ deficiency is illustrated in Figure 2.

### 3.2. Broader Impacts on Assisted Reproductive Technologies and on Reproductive Outcomes

The clinical relevance of PLCζ deficiency extends across assisted reproductive technologies (ART), including both intracytoplasmic sperm injection (ICSI) and conventional in vitro fertilization (IVF). Traditionally, PLCζ deficiencies have been associated with total fertilization failure (TFF) following ICSI [4,6]. However, recent studies have expanded this understanding to include implications for conventional IVF (cIVF). For instance, research by Che et al. (2023) identified specific mutations in the *PLCZ1* gene that were associated with polyspermy during cIVF. These mutations led to excessive sperm binding and delayed pronuclear formation, indicating a failure in the normal oocyte-activation process. Notably, a significant positive correlation was found between the proportion of sperm expressing PLCζ and fertilization success rates in cIVF [55]. Furthermore, a study by Tong et al. (2024) reported novel *PLCZ1* mutations in patients who experienced polyspermy during IVF cycles. The affected individuals exhibited failure of pronuclear formation during ICSI cycles, suggesting that *PLCZ1* mutations can lead to both polyspermy in IVF and fertilization failure in ICSI [56].

Regarding the mechanism behind fertilization failure and the effect of PLCζ on reproductive outcomes, PLCζ deficiency disrupts oocyte activation, causing the oocyte to remain arrested in metaphase II. Consequently, pronucleus formation is either delayed or fails entirely, preventing fertilization from proceeding. However, not every PLCζ mutation or deficiency completely abolishes oocyte activation; in some cases, a reduced PLCζ level or a hypomorphic variant may trigger delayed or incomplete activation. Specifically, when fertilization does occur despite low PLCζ levels, abnormal outcomes such as the formation of a single pronucleus, issues with embryonic development or irregular cleavage patterns are observed, reflecting incomplete or asynchronous egg activation [57]. Specifically, it has been observed that even if a globozoospermic sperm manages to fertilize an egg, the results of the fertilization are often abnormal (e.g., a single pronucleus), due to incomplete egg activation [58]. In animal models, researchers have observed that PLCζ-knockout male mice are subfertile rather than completely sterile—some eggs do fertilize, but with significant abnormalities. Notably, mouse eggs fertilized by PLCζ-null sperm showed delays in development and a high incidence of polyspermy [59]. This suggests that when PLCζ is absent or deficient, eggs may linger in a vulnerable state, allowing extra sperm to penetrate or failing to proceed through early mitotic divisions on time. By analogy, in human IVF, a sperm with mild PLCζ impairment might fertilize the egg but yield a poor-quality embryo that either arrests or leads to miscarriage. In a study by Che et al. (2023), the researchers propose a threshold, reporting that a sperm population with fewer than 57% PLCζ-positive cells is significantly associated with poor fertilization outcomes. They observed that in conventional IVF (cIVF), PLCζ-null spermatozoa can induce atypical Ca^2^^+^ spikes—of significantly lower amplitude and frequency—that are insufficient to trigger proper oocyte activation or to establish a block to polyspermy. As additional sperm fuse with the oolemma, the number of Ca^2^^+^ spikes increases, and multiple pronuclei may form once a threshold of intracellular Ca^2^^+^ signaling is reached, resulting in polyspermy and abnormal fertilization [55].

Clinically, this dysfunction may extend beyond fertilization, as emerging evidence links PLCζ deficiencies to recurrent implantation failure (RIF) and recurrent pregnancy loss (RPL). In couples with RIF/RPL for whom female factors have been ruled out, scrutiny often shifts to the male’s capacity for sperm activation. Recent analyses have included men with a history of RIF or early miscarriage in studies of PLCζ, revealing that some of these men exhibit lower PLCζ levels or abnormal localization patterns in their sperm [60,61]. The hypothesis is that even if fertilization occurs, a suboptimal Ca^2^^+^-oscillation regime could impair the egg’s full activation and the embryo’s developmental program. Early embryonic events such as genome activation, cleavage timing, and epigenetic remodeling might be disrupted, resulting in embryos that fail to implant or that implant but cannot progress. While direct evidence is still emerging, it can be speculated that reduced PLCζ levels may contribute to the formation of embryos with low developmental potential, manifesting as repeated IVF implantation failures or very early pregnancy losses. Supporting this, one study found that ICSI fertilization rates correlate with the proportion of sperm containing PLCζ—men with higher overall PLCζ levels in their sperm tended to achieve better fertilization and blastocyst development [23]. Conversely, if a man’s sperm population has broadly poor PLCζ expression, poor fertilization is observed [55], potentially leading to the formation of less viable embryos. More research is needed to firmly link PLCζ to RIF/RPL, but this line of inquiry underlines that PLCζ’s role does not end at fertilization; the quality of the calcium-oscillation signal can influence the trajectory of embryo development.

In summary, a spectrum of male fertility issues—from total fertilization failure to subtle problems in embryonic development—can be traced back to the level of PLCζ in sperm. Recognizing this expands the impact of PLCζ research by addressing not only the question “Why is there no fertilization?” but also “What is the threshold for fertilization?” and “Why does poor development occur even when fertilization happens?”.

## 4. Diagnostic Strategies

Given the critical role of PLCζ in oocyte activation and fertilization, the identification of deficiencies in its expression or function has become an important focus in the diagnostic evaluation of men. Traditional semen analysis may overlook functional defects such as PLCζ deficiency, which can exist even in normozoospermic men. Therefore, specialized diagnostic approaches are needed to assess PLCζ presence, localization, and activity, offering a more accurate picture of sperm fertilization potential and guiding clinical decision-making. Today, several diagnostic strategies are employed:

Immunofluorescence (IF) analysis of sperm PLCζ: This is the test most commonly used to evaluate PLCζ in a clinical setting. Sperm from the patient are fixed to slides and stained with a PLCζ-specific antibody, and fluorescence microscopy is used to visualize the PLCζ content and location in the sperm head [62]. A normal result would show a strong PLCζ signal (often a band in the equatorial/post-acrosomal region of the sperm head) in a high proportion of sperm. In contrast, patients with OAD typically exhibit either dramatically reduced fluorescence intensity or complete absence of PLCζ staining in their sperm [23]. The IF assay can also reveal abnormal localization patterns; for example, PLCζ may be predominantly found in the midpiece or may form punctate foci instead of a uniform equatorial distribution, patterns associated with poor oocyte-activation ability [46,63]. Quantitative immunofluorescence can estimate the percentage of PLCζ-positive sperm and the relative level of PLCζ per cell. In practice, this assay has become a valuable prognostic indicator: a low PLCζ score in a man’s sperm strongly correlates with fertilization failure [64]. It is worth noting that proper execution of the IF test is crucial. For this reason, some protocols use antigen-unmasking techniques to expose PLCζ epitopes and enhance detection [25]. Standardization of the staining and scoring method across laboratories is an ongoing effort. Nonetheless, IF provides a relatively accessible diagnostic readout of the sperm’s PLCζ status.

Western blot and protein quantification: In research or specialized diagnostic laboratories, a Western blot can be performed on a sperm sample to detect and quantify PLCζ protein. This process involves lysing a large quantity of sperm, running the proteins on a gel, and using an antibody against PLCζ to determine the presence and intensity of the protein band. Western blots have confirmed that men with fertilization failure often have significantly lower PLCζ protein levels compared to fertile controls [47]. For instance, the normozoospermic infertility patient mentioned earlier exhibited a faint PLCζ band at approximately 70 kDa with ~20% of the intensity seen in fertile men [65]. While Western blots provide more quantitative data than IF does, they require a substantial sperm count and do not indicate the proportion of sperm affected, as they analyze protein expression in bulk sperm lysates, providing an average protein level across the entire sample. Most importantly, however, Western blotting cannot provide information about the subcellular localization of PLCζ, which is crucial for PLCζ’s function in oocyte activation. Therefore, Western blots are typically used as adjunctive methods. Some studies also use ELISA-based assays or flow cytometry to quantitatively measure PLCζ levels in sperm populations [65]. Flow cytometry, for example, can measure the fluorescence of PLCζ staining across thousands of sperm, revealing significant reductions in PLCζ in men with abnormal semen parameters [66]. These quantitative approaches reinforce IF findings and help establish cut-off values for what constitutes “low PLCζ”.

Bioassays of oocyte-activation capacity: Functional tests can directly assess whether a man’s sperm can activate an oocyte. The classic assay is the mouse oocyte-activation test (MOAT), in which the patient’s sperm is injected into mouse eggs to determine whether Ca^2^^+^ oscillations or cleavage occur. If the sperm is PLCζ-deficient, the mouse egg typically fails to exhibit Ca^2^^+^ oscillations [5]. Consequently, no polar-body extrusion or pronuclear formation will happen in the mouse egg. Historically, MOAT has been used to implicate sperm in cases of infertility; for example, human sperm that consistently fail to activate mouse eggs almost certainly lack a functional activating factor [63]. A related approach, when available, is to use spare human oocytes (e.g., unfertilized eggs from IVF cycles) in a human oocyte-activation assay, often measuring Ca^2^^+^ [67]. In a controlled setting, the patient’s sperm can be microinjected into a donated human oocyte for monitoring of Ca^2^^+^ release; failure to observe oscillations would confirm that the sperm is the issue. While powerful, these bioassays are not routine, as they require fresh oocytes and specialized equipment. They are typically used for research or for confirming a diagnosis when other tests are inconclusive. Still, they provide the most direct evidence of PLCζ functional status—essentially a live readout of whether the sperm triggers Ca^2^^+^ spikes or not. These tests also help differentiate whether a fertilization failure in a couple is due to problems in the sperm or egg [5]. For instance, if a man’s sperm fails to activate even a donor egg, the problem lies with the sperm (and likely PLCζ); conversely, if it activates a donor egg, the issue may be with the female partner’s oocyte quality.

Genetic testing (*PLCZ1* gene mutations): Genetic testing for mutations in the *PLCZ1* gene has become an important diagnostic tool in cases of unexplained fertilization failure. If a PLCζ problem is suspected, sequencing the man’s *PLCZ1* gene can sometimes reveal pathogenic variants. Both Sanger sequencing and next-generation sequencing (NGS) can identify pathogenic variants, including missense, nonsense, or frameshift mutations, that impair PLCζ synthesis, stability, enzymatic activity, or localization. Several studies have reported homozygous and compound heterozygous *PLCZ1* mutations in men that disrupt the protein’s structure or functionality [26,43,68]. Genetic testing is particularly relevant in consanguineous families or recurrent cases [69]. For example, six novel *PLCZ1* mutations were reported in five families, all leading to the absence of PLCζ protein and subsequent fertilization failure; importantly, microinjection of wild-type PLCζ mRNA rescued activation in those cases, confirming causality [69]. Although routine genetic screening for *PLCZ1* is not yet standard practice, as the catalog of mutations and genotype–phenotype data grows, it could become a valuable diagnostic adjunct—potentially allowing for pre-ICSI counseling if a man is found to carry a *PLCZ1* defect. However, interpretation of genetic findings can be challenging, particularly when variants of uncertain significance are identified; in such cases, functional validation through additional assays may be necessary.

In practice, a combination of these approaches often yields the clearest diagnosis. For a couple with unexplained TFF, the man’s sperm can be analyzed via immunofluorescence for PLCζ. If results are borderline or equivocal, a functional test such as MOAT or a Western blot can provide additional evidence, and genetic testing might be considered if a hereditary issue is suspected. The clinical significance of diagnosing PLCζ deficiency is substantial: once confirmed, it informs the treatment plan (e.g., using assisted oocyte activation in future ICSI cycles or considering experimental therapies). It also spares the couple repeated failed cycles by addressing the core issue. Thus, PLCζ testing has become an important piece of the “male infertility puzzle”, helping to identify elusive sperm-factor issues that were historically diagnosed only by exclusion. An overview of these diagnostic strategies is summarized in Table 1.

## 5. Therapeutic Approaches

### 5.1. Assisted Oocyte Activation (AOA)

The most immediate therapeutic strategy for PLCζ-related fertilization failure is to bypass the need for sperm PLCζ by artificially activating the oocyte, a process known as assisted oocyte activation (AOA). In practice, once ICSI has been performed (sperm injected), the oocytes are exposed to a stimulus that elevates intracellular Ca^2^^+^, triggering the activation cascade that the sperm failed to induce [70,71]. Mechanical, electrical, and chemical stimuli are all used for AOA and are associated with unique risks and benefits [71].

The simplest and most widely used AOA method involves chemical activation with a calcium ionophore. These methods utilize lipid-soluble compounds that diffuse into the oocyte, enhancing calcium permeability and promoting both calcium influx from the extracellular environment and the release of calcium from intracellular stores [72]. Ionophores such as calcimycin (A23187) or ionomycin can be added to the culture medium for a brief period, mimicking the effect of PLCζ. Although Ca^2+^ ionophores elicit single Ca^2+^ transients in oocytes and do not replicate the physiological calcium release observed during normal fertilization, where multiple calcium oscillations occur, they are highly effective in triggering oocyte activation. Numerous case reports and series have demonstrated that ionophore-based AOA can rescue fertilization in cases of PLCζ deficiency [23,72]. For example, in the case of the normozoospermic man with absent PLCζ, applying a calcium ionophore to his partner’s oocytes after ICSI led to normal fertilization and subsequent embryo development, whereas without AOA, none of the oocytes would fertilize [52]. Larger studies have also shown that AOA significantly improves fertilization rates and even pregnancy rates for couples with a history of failed or low fertilization [73]. One report indicated that across patients with prior fertilization failure, the fertilization rate per oocyte increased from ~25% to ~48% in subsequent cycles in which calcium ionophore AOA was used [74]. Importantly, these AOA-conceived embryos can develop into blastocysts and lead to healthy live births, indicating that the artificial stimulus can effectively substitute for the sperm factor. In addition to ionophores, strontium chloride (SrCl_2_) has garnered interest as an activating agent. SrCl_2_ is widely regarded as the most efficient method for inducing oocyte activation in mouse oocytes, as it produces multiple Ca^2+^ transients and leads to high rates of blastocyst formation [75,76]. However, the mechanism of action of strontium chloride in humans remains unclear [77]. Another method used in research is ethanol exposure, which also elicits a single rise in Ca^2+^ and appears to increase the rate of formation of high-quality embryos and blastocysts [78]. Besides chemical methods, mechanical activation techniques can also be employed. One common approach involves repeated cytoplasmic aspiration using the ICSI pipette, which is thought to disrupt the oocyte’s cytoskeleton and membrane integrity in a manner that facilitates intracellular Ca^2^^+^ release [79]. While this technique avoids chemical exposure, it is technically demanding, requiring careful manipulation to avoid oocyte damage. Some clinics or laboratories use also electrical pulses (electroactivation) to transiently permeabilize the oocyte and release Ca^2^^+^ from the ER [76,80].

The choice of AOA method often depends on laboratory protocols, clinician preference or experience, and local regulatory approvals. Among the available options, calcium ionophores have been the mainstay in human IVF due to familiarity, efficacy, and the availability of data on relative safety. Regardless of the approach, all these methods share the goal of initiating one or more Ca^2^^+^ rises in the egg to jump-start the activation process. However, substantial variability exists between methods, and their effectiveness can differ based on both biological and technical factors. For instance, ionomycin has been shown to induce the release of more intracellular Ca^2^^+^ and lead to better fertilization rates than calcimycin in both mouse and human oocytes [81]. These results are consistent with previous findings suggesting that oocyte activation is triggered when the total dose of Ca^2+^ reaches a minimal threshold [82]. Consequently, the choice of chemical agent, along with the activation protocol (including concentration, timing, and duration of exposure), directly influences the total amount of Ca^2^^+^ discharged. This may account for the variability in efficacy reported across studies and help explain inconsistent outcomes found in some AOA trials [72].

Clinically, AOA is applied judiciously. It is generally indicated for couples who have experienced TFF or consistently low fertilization rates attributable to sperm-related OAD, such as confirmed low levels of sperm PLCζ. In these cases, AOA can be incorporated into ICSI procedures for all oocytes as a preventative measure. The technique has enabled many such couples to achieve pregnancies in situations where standard ICSI failed. For example, in a series of men with PLCζ mutations, the use of AOA in their ICSI cycles successfully restored normal fertilization in the majority of oocytes [69]. However, because AOA involves extra manipulation of the oocyte, it is not used unless necessary. Nevertheless, when indicated, it is a powerful tool. AOA has evolved from an experimental intervention to an accepted adjunct in cases of repeated fertilization failure. Current evidence, including follow-up studies of children born, indicates that AOA does not have obvious adverse effects on offspring [73]. However, given that AOA bypasses a physiological step in fertilization and involves artificial stimulation of oocyte activation, long-term safety data are still limited and continued monitoring of offspring remains essential.

In summary, AOA, especially with calcium ionophores, is the frontline therapy for couples suffering from PLCζ-related infertility. It revolutionizes the management of these cases by allowing embryologists to actively induce activation and usually rescue the cycle, rather than subject patients to repeated failures. The success stories associated with AOA underscore how understanding PLCζ’s role has directly contributed to effective clinical interventions. Nonetheless, AOA is a workaround; it does not fix the underlying sperm defect, and it introduces artificial steps into the process of conception. This has driven interest in more targeted remedies that focus specifically on PLCζ.

### 5.2. Emerging Treatments: PLCζ Protein/mRNA Therapy and Novel Interventions

While AOA has been a game-changer, it is essentially a generic fix, as it does not utilize the sperm’s own activator. Emerging therapies aim to supplement or replace PLCζ more directly. One approach under active research is the use of recombinant human PLCζ protein as a therapeutic agent. This method aims to restore the natural activation pathway by providing a functional version of the missing or defective sperm factor. Recently scientists have successfully produced recombinant PLCζ in the lab by expressing the human *PLCZ1* gene in bacteria and purifying the protein, simultaneously addressing challenges related to stability and biological activity that had previously limited progress [83]. A recent breakthrough reported the development of a stable, active recombinant PLCζ that retains full enzymatic function for months when stored [83]. When this purified PLCζ was microinjected into mouse oocytes, it induced normal Ca^2^^+^ oscillations comparable to those caused by natural sperm [83]. Remarkably, experiments showed that microinjecting recombinant PLCζ into eggs resulted in higher rates of blastocyst formation than did traditional calcium ionophore-based activation [84]. This suggests that using PLCζ protein could provide a more physiologically balanced activation stimulus, as it generates a series of calcium oscillations rather than a one-off Ca^2^^+^ spike. Furthermore, early safety tests of recombinant PLCζ have been encouraging—for instance, exposing developing chicken embryos to the PLCζ protein did not impair their viability [83], suggesting no overt toxic effects. Before human use, however, recombinant PLCζ will require rigorous purification, activity testing, and validation to ensure it does not introduce contaminants or cause an immune response. Nevertheless, the concept holds great promise as a targeted remedy for male infertility: adding back the missing sperm factor.

Another cutting-edge strategy is PLCζ mRNA therapy. Instead of providing recombinant PLCζ protein, this approach involves microinjecting synthetic mRNA encoding PLCζ, which the oocyte can then translate into a functional protein. The oocyte is an ideal system for this because it is translationally active and large enough to handle microinjections of mRNA. Proof of concept comes from both animal and human studies. In a key experiment, human oocytes that had failed to fertilize were injected with synthetic wild-type PLCζ mRNA, resulting in activation and subsequent blastocyst development in a substantial fraction of the eggs [85]. Specifically, around 64.6% of the mRNA-injected human oocytes showed normal fertilization (formation of two pronuclei), and approximately 35% developed to the blastocyst stage [85]. In contrast, oocytes injected with an infertile patient’s mutant PLCζ mRNA (for example, the I489F mutation) largely failed to activate, with only ~14% achieving fertilization and none reaching the blastocyst stage [85]. This elegantly demonstrated that it is possible to “rescue” human oocyte activation by supplying the correct PLCζ transcript and that the problem in the patient’s sperm was definitively due to the mutant PLCζ. mRNA injection has also some practical advantages: RNA is often easier to produce and handle than protein in some ways, and a very small amount can be enough to generate the protein inside the oocyte. However, there are also challenges, such as ensuring the mRNA is optimally translated and does not trigger any anti-viral RNA sensors in the oocyte. That said, mammalian oocytes have been successfully activated with PLCζ mRNA in several research contexts [85]. Furthermore, delivering PLCζ mRNA poses another challenge, with lipid nanoparticle-based systems being investigated for their efficiency and safety [86].

The development of therapies involving supplementation of PLCζ, whether via protein or mRNA, is a prime example of bench-to-bedside innovation. It arises directly from identifying PLCζ as the oocyte-activation factor and understanding its mechanism. By using this genuine factor, these approaches aim to replicate the natural physiological process more closely than a brute-force approach using a Ca^2^^+^ ionophore. This more physiological activation may lead to improved embryonic development, and by more accurately mimicking the natural activation process, PLCζ therapy may carry a reduced risk of epigenetic abnormalities in the resulting embryos, although this requires confirmation in long-term studies. These therapies could be also especially valuable in cases in which AOA might work inconsistently or in which the oscillation quality is linked to embryo quality. Nevertheless, these therapies are still in the experimental stages, and there are important regulatory and ethical considerations to address. Over-injecting PLCζ can lead to an abnormal frequency and amplitude of Ca^2+^ oscillations, resulting in low rates of blastocyst development [84]. Conversely, the dose of injected RNA may be insufficient for translation into enough PLCζ to trigger Ca^2+^ influx or release from the ER, potentially causing abnormal Ca^2+^ release [72]. Furthermore, introducing a recombinant protein or exogenous mRNA into a human oocyte would likely be classified as advanced manipulation by fertility regulators. This intervention constitutes a form of germline manipulation at the single-generation level, as it acts at a critical stage of early embryonic development. Clinical trials will be needed to ensure safety, specifically addressing questions such as whether the exogenous PLCζ confines its action to the intended egg without affecting later embryonic gene expression and whether it increases the risk of imprinting errors. So far, initial studies—like tests in mouse and chicken embryos—have been reassuring [83], but extensive validation is still required.

In summary, therapy for PLCζ deficiencies is evolving from an indirect approach (AOA) to direct replacement therapy (PLCζ protein or mRNA). Early results are very promising, suggesting that we may one day routinely inject a PLCζ solution alongside sperm for certain patients. The focus is always on safety and efficacy—providing just enough activation without causing harm. Regulatory agencies will scrutinize these emerging therapies; however, given the success of AOA and the clear need, it is likely that with careful trials, PLCζ-based therapies will become a reality. These interventions would specifically address the root cause of infertility (the missing or defective sperm factor), representing a more “curative” approach than the workaround that AOA provides.

## 6. Future Directions

Research on PLCζ is rapidly progressing, and several key areas are poised to enhance both the scientific understanding and clinical management of male infertility. The following areas represent major avenues for future research and clinical development aimed at improving the management of PLCζ-related infertility:

Standardization of PLCζ testing: As discussed, diagnosing PLCζ deficiency currently relies on lab-developed assays, such as immunofluorescence and Western blotting, which can vary between centers due to the lack of a universally accepted standard for PLCζ testing in clinical practice. A crucial future step is to standardize these diagnostic methods. This includes developing calibrated reference materials, such as purified PLCζ protein, to quantify assay sensitivity and consensus protocols for immunostaining and image analysis. For instance, antigen-unmasking techniques have been shown to improve PLCζ visualization [6], but not all labs use them. Standardization would ensure that a “low PLCζ” result in one clinic has the same meaning in another. Additionally, studies should aim to define clear cutoff values at which a PLCζ level should be considered insufficient for fertilization. Large-scale correlations of PLCζ quantification with fertilization outcomes will help to establish these thresholds [23], enabling more consistent patient selection for AOA or future PLCζ-based therapies. It has also been observed that PLCζ levels can vary even within different ejaculates from the same man [67], potentially due to sampling or physiological fluctuations. Future diagnostic practices might incorporate multiple semen samples or an average to get a true picture of a man’s PLCζ status. Automating the analysis (e.g., using flow cytometry or computer vision on stained sperm images) could enhance objectivity. All these efforts will help make PLCζ deficiency a more routinely recognized diagnosis. The ultimate goal is to develop an accredited test kit for PLCζ—potentially an ELISA or a standardized immunofluorescence slide—that can be made available to IVF labs, one similar to kits that currently exist for other infertility biomarkers.

Long-term safety of AOA and offspring health: While short-term studies of assisted oocyte-activation outcomes are reassuring, ongoing vigilance is necessary as more children conceived with AOA grow up. Thus far, data indicate no significant increase in birth defects or developmental delays in children born from ICSI–AOA compared to conventional ICSI [73]. A 2020 meta-analysis of five studies found the birth-defect rate after AOA was statistically no different than that after conventional ICSI (risk ratio ~1.27, which was not significant) [73]. This serves as important evidence supporting the safety of AOA. Nonetheless, these studies focus primarily on early childhood; it will be valuable to follow these cohorts into later childhood and adolescence to detect any possible subtle effects, such as those on offspring fertility, neurological development, or metabolic and immunological health. Epigenetic patterns established at fertilization could, in theory, be altered by differences in Ca^2^^+^ signaling. Therefore, comprehensive and longitudinal follow-up studies are essential. These studies should monitor not only physical health outcomes, but also potential epigenetic changes and reproductive health in the next generation. So far, results are largely positive, but maintaining registries of AOA cases and continuing comparative studies will strengthen the safety profile. Furthermore, designing large, multicenter registries to systematically collect standardized outcome data will be crucial for detecting any rare or delayed adverse effects. If any concerns arise, they will guide refinements in technique, such as adjusting ionophore exposure time or concentration. Overall, the data suggest AOA is safe, but the commitment to “first do no harm” means this will remain an area of active surveillance.

Understanding testicular regulation of PLCζ expression: Another future research direction will involve unraveling why some men have low PLCζ levels in their sperm. Beyond genetic mutations in *PLCZ1*, there may be upstream regulatory factors to consider. Research could explore questions such as “Are there transcription factors during spermatogenesis that drive *PLCZ1* expression?”, “Do hormonal levels (testosterone, FSH) influence the amount of PLCζ a developing spermatid accumulates?”, and “Are there conditions (e.g., high fever, toxins, varicocele) that specifically impact PLCζ production or retention in sperm?”. The observation that varicocele patients have reduced PLCζ leveks suggests that oxidative stress in the testes can reduce PLCζ mRNA and protein levels [54]. This raises the possibility that improving the testicular environment (e.g., through varicocele repair or antioxidant therapy) before IVF could improve PLCζ content in sperm, thereby enhancing fertilization potential naturally. Future studies might test interventions, such as providing an infertile man with supplements (like certain antioxidants or micronutrients) to see if his sperm PLCζ levels increase. Moreover, the process by which PLCζ is packaged into the sperm’s perinuclear theca during spermiogenesis is still being elucidated. It likely involves other proteins (like PAWP/WBP2NL and CAPZA3) that have been co-implicated in oocyte activation. Interestingly, one study found a correlation between *CAPZA3* and *PLCZ1* expression in men—hinting at a coordinated pathway in sperm development [87]. Investigating such pathways could reveal targets for boosting PLCζ loading into sperm. In the future, we might even see pharmacological upregulation of PLCζ as a treatment—if, for instance, a certain signaling pathway in spermatogenesis that enhances PLCζ could be safely modulated. Overall, gaining insight into the spermatogenic control of PLCζ will enrich our ability to prevent and treat OAD at its root.

Considerations for gene therapy and germline editing: Looking ahead, beyond protein or mRNA supplementation, future strategies may involve direct correction of PLCζ gene defects through gene therapy. For example, one could envision using CRISPR/Cas9 genome editing on a patient’s spermatogonial stem cells to repair pathogenic mutations, then using these corrected cells to generate functional sperm (in vivo or in vitro). While this may currently seem like science fiction for humans, similar approaches are already being attempted in animal models for other genetic defects. At present, ethical guidelines prohibit gene editing for infertility in humans. However, the ethical landscape may evolve, especially if interventions involve targeting somatic precursor cells, such as spermatogonial stem cells, rather than directly editing a fertilized embryo. There is also the prospect of creating induced pluripotent stem cells (iPSCs) from the patient, correcting the mutation in vitro, and subsequently deriving functional gametes. The implementation of these ideas is still far off; any clinical application would require overcoming technical issues and achieving societal consensus. Nonetheless, they represent a notable future direction as they offer a potential cure, restoring man’s natural fertility without the need for assisted reproduction. However, these approaches raise significant ethical questions and technical hurdles, as germline gene editing would affect future generations. For now, while they remain on the distant horizon, they highlight the transformative potential of ongoing research into the molecular underpinnings of male infertility.

Function-based sperm selection and novel IVF lab techniques: In the nearer term, IVF laboratories might implement advanced sperm-selection methods aimed at maximizing the chances of using sperm capable of triggering oocyte activation. One technique that has already been tested in globozoospermia is high-magnification sperm selection through motile sperm-organelle-morphology examination (MSOME). In globozoospermic samples, where most sperm are defective, MSOME allows embryologists to select the few spermatozoa exhibiting partial acrosome formation and potentially higher PLCζ levels for ICSI. Indeed, one study found that selecting globozoospermic sperm with the best shape (via MSOME) enriched for those with higher PLCζ, leading to better fertilization outcomes [23]. A promising concept under exploration is the assessment of “PLCζ content” as a sperm-selection criterion. While we cannot yet live-stain sperm for PLCζ without compromising their viability for ICSI, indirect methods are emerging. The Zeta potential method is one such method; it utilizes the charge properties of the sperm membrane, which correlate with sperm maturity and potentially PLCζ content. Some laboratories already use a Zeta-potential filter to select sperm, though data on PLCζ enrichment are limited. Microfluidic technologies also offer exciting possibilities, as they can sort sperm based on motility and potentially based on surface markers without causing damage. Future devices might be able to separate sperm subpopulations in a semen sample, and if these can be correlated with high PLCζ (for example, perhaps the most mature, morphologically normal sperm have the highest PLCζ), then simply choosing those sperm for ICSI could improve fertilization outcomes [23]. For instance, a chip could measure each sperm’s ability to mobilize Ca^2^^+^ in a tiny drop or bind those with a certain surface charge related to PLCζ presence. Additionally, the development of non-invasive imaging technologies, such as Raman spectroscopy or advanced forms of live-cell microscopy, could revolutionize sperm selection by enabling real-time assessment of molecular markers like PLCζ in viable sperm. While these techniques are speculative, the goal is known: to select the “best” sperm not only by appearance and motility, but also by functional competence at the molecular level.

Refinement of PLCζ-based therapies: As techniques involving recombinant PLCζ protein and mRNA advance, there will be a need to refine dosage and delivery. Future research will likely focus on dosing studies answering questions such as “How much PLCζ protein or mRNA is optimal to inject per oocyte?” and “What timing yields the best oscillation pattern?”. Achieving the correct therapeutic dose is essential: insufficient levels of PLCζ may fail to initiate proper calcium oscillations and oocyte activation, whereas excessive amounts could lead to abnormal or dysregulated calcium signaling, potentially impairing fertilization or early embryonic development. For PLCζ protein therapy, key challenges include maintaining protein stability during storage and handling and ensuring functional activity at the time of microinjection. For mRNA-based therapy, additional variables come into play. Synthetic mRNA must be efficiently delivered into the oocyte cytoplasm, remain stable long enough to allow translation, and produce physiologically appropriate levels of PLCζ protein. Advances in mRNA engineering—including optimized untranslated regions (UTRs), codon optimization, and chemical modifications—could enhance translation efficiency and stability while minimizing immunogenicity. There is also interest in making PLCζ delivery more convenient, such as by possibly attaching PLCζ to the sperm itself (e.g., a “loaded” sperm where PLCζ protein is artificially bound to it before ICSI). These kinds of innovations will be explored to simplify the workflow. Furthermore, regulatory approval for PLCζ protein use will require the demonstration of consistent manufacturing and the absence of adverse effects. If those hurdles are overcome, PLCζ injection could become an available treatment in the IVF arsenal.

In essence, the future of PLCζ research is two-pronged: It will involve both (1) deepening our understanding of its biology—examining how PLCζ is regulated, how it interacts with other sperm factors, and its role in embryo development; and (2) enhancing the clinical toolkit—improving diagnostics and developing safer, more effective therapies for PLCζ-related infertility. The story of PLCζ has been one of rapid translation from discovery to application, and ongoing bench-to-bedside efforts will continue to bridge existing gaps. The ultimate vision is to ensure that no couple experiences unexplained fertilization failure—a quick sperm PLCζ test will identify the issue, and a targeted remedy (like adding PLCζ or using an optimized AOA protocol) will help them achieve fertilization and the growth of healthy embryos.

## 7. Conclusions

Phospholipase Cζ has proven to be a pivotal molecule at the nexus of fertilization and male infertility, truly living up to its name as the sperm’s “molecular spark” for new life. This sperm-specific enzyme is now recognized as the essential trigger that wakens the oocyte from dormancy, and its discovery has resolved long-standing questions about certain fertilization failures. Over the last decade, research spanning in focus from basic biochemistry to clinical IVF has built a cohesive narrative: when PLCζ is present and functional, fertilization proceeds normally, but when PLCζ is absent or faulty, the egg remains inert and fertilization (or further embryogenesis) can falter. This understanding has immense translational value. Clinicians can now diagnose oocyte-activation deficiency by checking the sperm’s PLCζ levels, providing clear answers to couples who previously had only guesses as to why their eggs did not fertilize. Therapies such as assisted oocyte activation, informed by PLCζ research, have transformed many IVF failures into successes. Looking ahead, the field is moving toward even more precise interventions, such as potentially administering PLCζ itself to an oocyte to restore the natural fertilization pathway. Each advance underscores the value of cross-disciplinary collaboration: biochemists, geneticists, embryologists, and clinicians are all working together to address the problem of male-factor fertilization failure.

In conclusion, the tale of PLCζ is a shining example of bench-to-bedside progress. What began as a hunt for a mysterious sperm factor has culminated in tangible clinical practices that improve patient outcomes. Yet, the story is not over. Ongoing research and innovation will continue to refine our understanding of this pathway—ranging from standardizing diagnostic assays and ensuring the safety of AOA-born children, to potentially preventing activation failure by enhancing PLCζ during spermatogenesis or repairing it through genetic means. The insights gained from PLCζ research extend beyond a rare subset of patients; they deepen our fundamental understanding of fertilization, embryonic development, and the intricate dance of gametes. Ultimately, the knowledge that “it takes two to spark an egg to life—an oocyte and a sperm with PLCζ” has powerful implications. It underscores that male fertility is not solely about delivering DNA, but also about providing an activation signal. As we harness this knowledge, reproductive medicine can continue to evolve, ensuring that the absence of this molecular spark does not extinguish a couple’s hopes of conception. The journey of PLCζ from bench to bedside exemplifies the translation of molecular science into the miracle of life, holding promise for many future innovations in the realm of fertility treatment.

## Figures and Tables

**Table 1:** Diagnostic approaches for identifying PLCζ deficiency in human sperm.

Method	Key Principle/ Procedure	Main Observations/Points	References
Immunofluorescence (IF)	- Patient sperm are fixed onto slides and labeled with a specific anti-PLCζ antibody. - Fluorescence microscopy is used to detect signal.	- Most commonly used clinical test. - Reveals whether PLCζ is present, reduced, or mislocalized. - Low or absent signal correlates with fertilization failure (TFF). - Some labs use antigen unmasking.	[23,25,46,62,64]
Western Blot/Protein Quantification (e.g., Flow Cytometry)	- Sperm proteins are extracted and separated (Western blot). - Antibody to PLCζ detects the specific band. - Flow cytometry measures PLCζ levels across many sperm.	- Can confirm significantly reduced PLCζ in men with recurrent TFF despite normal semen parameters. - Quantifies protein amount but does not show subcellular localization. - Requires ample sperm.	[47,65,66]
Functional Oocyte-Activation Tests (e.g., Mouse Oocyte Activation Test)	- Patient’s sperm are microinjected into mouse oocytes (or spare human oocytes) to assess whether they trigger Ca^2^^+^ oscillations and pronuclear formation.	- Considered a “gold-standard” functional assay. - If sperm lack functional PLCζ, no or minimal Ca^2^^+^ release occurs. - Not routinely available due to ethical and technical constraints.	[5,63,67]
Genetic Testing (PLCZ1 Gene Sequencing)	- Screening for pathogenic variants or deletions in the PLCZ1 gene (via Sanger or next-generation sequencing).	- Identifies causative mutations in some patients with unexplained TFF. - Useful in consanguineous families or repeated fertilization failures. - Not yet standard; variants require functional validation.	[26,42,43,44,45,68,69]

PLCζ, phospholipase C zeta; TFF, total fertilization failure; Ca^2^^+^, calcium ions.

## Data Availability

No new data were created or analyzed in this study. Data sharing is not applicable to this article.
